# Rest-Mediated Regulation of Extracellular Matrix Is Crucial for Neural Development

**DOI:** 10.1371/journal.pone.0003656

**Published:** 2008-11-06

**Authors:** Yuh-Man Sun, Megan Cooper, Sophie Finch, Hsuan-Hwai Lin, Zhou-Feng Chen, Brenda P. Williams, Noel J. Buckley

**Affiliations:** 1 Centre for the Cellular Basis of Behaviour (CCBB), The James Black Centre, Institute of Psychiatry, King's College London, London, United Kingdom; 2 Departments of Anesthesiology, Psychiatry, and Developmental Biology, Washington University School of Medicine Pain Center, Saint Louis, Missouri, United States of America; Katholieke Universiteit Leuven, Belgium

## Abstract

Neural development from blastocysts is strictly controlled by intricate transcriptional programmes that initiate the down-regulation of pluripotent genes, *Oct4*, *Nanog* and *Rex1* in blastocysts followed by up-regulation of lineage-specific genes as neural development proceeds. Here, we demonstrate that the expression pattern of the transcription factor *Rest* mirrors those of pluripotent genes during neural development from embryonic stem (ES) cells and an early abrogation of Rest in ES cells using a combination of gene targeting and RNAi approaches causes defects in this process. Specifically, Rest ablation does not alter ES cell pluripotency, but impedes the production of Nestin^+^ neural stem cells, neural progenitor cells and neurons, and results in defective adhesion, decrease in cell proliferation, increase in cell death and neuronal phenotypic defects typified by a reduction in migration and neurite elaboration. We also show that these Rest-null phenotypes are due to the dysregulation of its direct or indirect target genes, *Lama1*, *Lamb1*, *Lamc1 and Lama2* and that these aberrant phenotypes can be rescued by laminins.

## Introduction

During mouse embryo development, the blastocyst differentiates into pluripotent primitive ectoderm and gives rise to a structure known as the epiblast [1]. The epiblast responds to extrinsic signals and generates three primary germ layers (ectoderm, mesoderm and endoderm) [2]. During neurulation, the ectoderm gives rise to the neuroectoderm in the form of a neural plate, which subsequently folds to generate the neural tube, composed of a single layer of neuroepithelial cells or neural stem cells (NSCs), where a series of ring-like constrictions mark the boundaries between the primordia of the major brain regions [3]–[4]. This process of neural development is orchestrated and accompanied by wholesale changes in transcriptional programmes and patterns of gene expression. However, due to the difficulties in accessing and manipulating early embryos, the transcriptional network that regulates neural development is poorly understood, especially in mammals. Embryonic stem (ES) cells derived from blastocysts retain the ability to recapitulate neural development *in vitro*, and offer an invaluable model to study early events in embryogenesis. The RE1 Silencing Transcription Factor / Neuron Restrictive Silencer Factor (Rest/Nrsf) is a zinc finger transcription repressor that has been postulated to act as a master regulator of neuronal gene expression in both the developing and mature nervous systems [5]–[6]. We and others have shown that Rest is highly expressed in blastocysts and ES cells, but that expression decreases as neural development proceeds [7]–[8]. In fact, down-regulation of Rest has been proposed to be obligate for differentiation of neural progenitors [8] and more recently, it has been proposed that Rest haplodeficiency results in loss of pluripotency markers and a reciprocal gain in differentiation markers [9]. Taken together, these observations suggest that Rest may play a crucial role at several stages of neural development. Here, we determine the function of Rest during neural development from ES cells through NSCs and neural progenitor cells (NPCs) to mature neurons using an *in vitro* ES cell-derived neural differentiation model.

Rest exerts its function by binding to both canonical and non-canonical RE1-sites identified at over 2000 loci in the mammalian genome [10]–[11] and is implicated in the regulation of both coding and non-coding genes [10], [12], many of which represent neuron-specific transcriptional units. The observation that many of these target genes are expressed by differentiated neurons, including ion channels, neurotransmitter receptors, neurotrophins, synaptic vesicle associated proteins, cell adhesion molecules, growth-associated and cytoskeletal proteins, gave rise to the initial perception that Rest acted as a silencer of neuron-specific genes in NPCs and non-neural cells to prevent precocious expression of neuronal characteristics. However, recent studies emerge that Rest has more versatile roles and can regulate its target genes either by activation, repression or silencing, depending upon the developmental stage and cell type [7], [13]. Rest recruits multiple cofactors, histone modifying and chromatin remodelling activities, all of which underwrite the complexity of Rest activity [14]–[16]. The diverse roles of Rest have been shown in both neural and non-neural pathologies including Huntington's disease, cardiac hypertrophy, medulloblastoma, malignant rhabdoid tumor, small cell lung cancer, ovarian cancer, and ischemia (see review for references [13]).

Despite the wealth of knowledge in identifying target genes [10]–[12] and in delineating the mechanistic actions of Rest [14]–[16], the biological function of Rest during neural development remains unclear. *Rest−/−* mice die around embryonic day (E)11.5, with embryo degeneration, neural tube malformations and widespread apoptosis evident from E9.5 [6]. Constitutive expression of Rest in chick spinal cord does not cause defects in neurogenesis but does result in axon pathfinding errors [17]. However, in Xenopus, disruption of Rest function disturbs ectoderm patterning and expands the neural plate [18], suggesting that Rest is indeed required for normal neural plate formation and neurogenesis. Collectively, these studies paint a somewhat ambiguous picture of the role of Rest in the development of NSCs and neurons. We have sought to address this issue by using a combination of gene targeting and RNAi to create ES lines expressing a range of Rest concentrations, which we have used to investigate the effect of Rest deficiency during ES cell-derived neural development. Importantly, in contrast to a recent study [9], we find that deletion of a single *Rest* allele does not result in any change in neural differentiation. Instead, we find that Rest levels have to be decreased by more than 92% to precipitate any phenotype. Rest ablation impairs the extracellular matrix (ECM) components and impedes the production of Nestin^+^ NSCs, NPCs and neurons. Furthermore, neurons derived from REST-null ES cells are devoid of elaborate processes, have defects in migration and undergo increased cell death. Importantly, all of these phenotypic effects of Rest ablation were rescued by treatment with laminins, a key component of the ECM that has been implicated in neuronal migration and more latterly in development of the neural plate. We propose a novel mechanism by which Rest regulates development of both NSCs and mature neurons by controlling expression of key components of the ECM.

## Results

### *Rest* expression during NSC and Neuron Development

**Figure 1:**
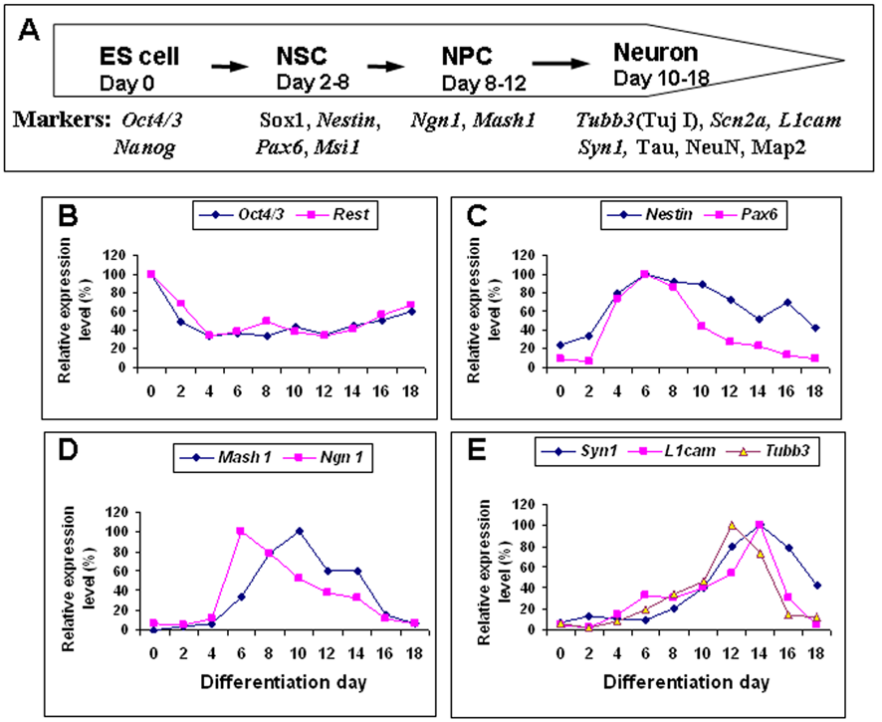
Time course of stage-specific marker expression during neural differentiation of HM1 embryonic stem (ES) cells. (A) Summary of neural stage-specific markers used in this study. NSC: neural stem cells; NPC: neural progenitor cells. (B) Down-regulation of *Oct4* and *Rest* was observed as neural differentiation proceeded. (C) From day 2–8, the expression of NSC markers, *Nestin and Pax6* is observed. (D) NPC markers, *Mash1* and *Ngn1*, appeared in an overlapping but slightly later wave than NSC markers. (E) After 10 days of differentiation, markers of early (*Tubb3*) *and* mature neurons (*Syn*1 and *L1cam*) appeared.

We investigated the role of Rest in neural development using an *in-vitro* ES cell-derived neural differentiation model, which recapitulates events during neural development in vivo. ES cells firstly differentiate into neuroepithelial cells (early NSCs), which peak around 4–6 days of differentiation and express Sox1 and Nestin (about 80% of population), and then differentiate further into more restricted NSCs that peak around 10 days of differentiation and express either Ngn1 or Mash1 (about 80% of population) (Fig. 1C–D and Fig. S2B–C). In this paper, we refer to early NSCs as NSCs and late more restricted NSCs as NPCs. To establish the time course during which NSCs, NPCs and neurons are formed from HM1 and 46C ES cells, we examined gene expression patterns for NSC markers (*Nestin* and *Pax6*), NPC markers (*Mash1* and *Ngn1*) and neuronal markers (*Tubb3*, Syn*1* and *L1cam*) during ES cell-derived neural differentiation (Fig. 1A). We found that *Rest* expression mirrored that of the pluripotent ES cell marker *Oct4*, and was expressed at highest level in ES cells with its expression level declining as differentiation proceeded; reaching its lowest level 4 days after differentiation just before NSC production reached its peak (Fig. 1B and Fig. S2A). *Rest* levels were maintained at low levels throughout neuron formation. Conversely, the expression patterns of *Nestin* and *Pax6* reciprocated those of *Rest* and *Oct4* indicating that NSC production started 1- or 2-days after differentiation, reached a peak around 6 days, and thereafter declined (Fig. 1C and Fig. S2B). Mash1 and *Ngn1* expression indicated that NPCs started to be produced after 6-days of differentiation and peaked between 6 and 10 days (Fig. 1D and Fig. S2C). The early neuronal marker *Tubb3* was observed around the same time but did not peak until 12 days of differentiation whereas the peak of mature neuron marker expression (*Syn1* and *L1cam*) occurred at 14–16 days of differentiation (Fig. 1E and Fig. S2D). This time course closely recapitulates the sequential generation of NSCs, NPCs and neurons observed *in vivo* (Fig. 1A).

### Rest ablation inhibits development of NSC and NPC

**Figure 2:**
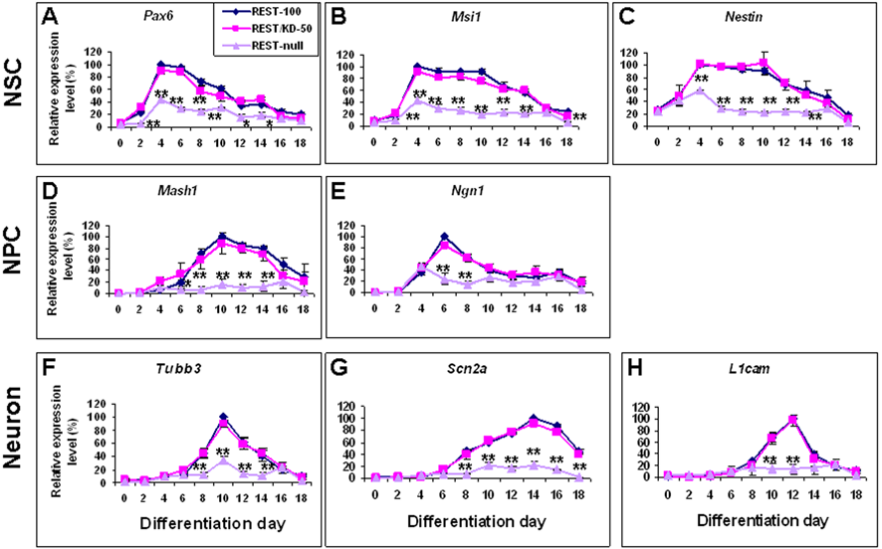
The effect of Rest ablation on the expression of stage-specific neural markers. The ability of control (REST-100) and Rest mutant ES cells (REST/KD-50 and REST-null) to generate the different neural cell populations was assessed by real time-PCR using the following markers: NSCs *Pax6*, *Msi1* and *Nestin* (A–C); NPCs *Mash1 and Ngn1* (D–E); early neurons *Tubb3* (F) and mature neurons, *Scn2a* and *L1cam* (G–H). Data are represented as mean±SEM. *P<0.05 and **P<0.01, significantly different from REST-100 and REST/KD-50.

Next we examined the effects of Rest ablation on the development of NSCs and NPCs. The control REST-100, REST/KD-50 and REST-null ES cells, which express 100%, 50% and 0% wild-type Rest levels respectively (Fig. S1B and S1D), were differentiated into NSCs and then into NPCs identified using an array of NSC and NPC markers (Fig. 1A). We assessed the effects of Rest ablation on gene and protein expression of these markers using quantitative Real-time PCR, FACS and immunocytochemical analysis. In REST-100 ES cells, the expression profiles of *Pax6*, *Msi1 and Nestin* were similar to one another, with expression peaking around 4 days and thereafter gradually declining (Fig. 2A–2C). REST/KD-50 ES cells showed no significant difference in the expression level and pattern of these genes as compared to the control REST-100 cells (Fig. 2A–2C). Similarly, no change in the number of Sox1^+^/Nestin^+^ NSCs was seen (Table 1 and Fig. S3B–3C). However, in REST-null ES cells, the peak expression of *Pax6* and *Msi1* was significantly (P<0.01) reduced to 40% of control levels whilst expression of *Nestin* was reduced to 60% of control levels (Fig. 2A–2C). This mutant generated significantly (P<0.01) fewer Sox1^+^/Nestin^+^ NSCs (52%) as compared to the REST-100 (77%) and REST/KD-50 ES cells (76%), but generated double the number of Sox1^+^/ Nestin^−^ NSCs compared with the control cells (P<0.01) (Table 1 and Fig. S3D). Moreover, REST-null ES cells produced lower levels of *Mash1* and *Ngn1* expression (P<0.01) and this was reflected in a parallel reduction in the number of Mash1^+^ NPCs (∼50%) as compared to the control (87%) (P<0.05) (Fig. 2D–2E; Table 1; Fig. S4B and 4D). Our results suggest that a 50% depletion of Rest shows no discernible effect on the production of NSCs and NPCs from ES cells. In fact, the production of early Sox1^+^/Nestin^−^ NSCs from REST-null ES cells remained unaffected but formation of late Sox1^+^/Nestin^+^ NSCs and subsequent production of NPCs was inhibited.

**Table 1:** The summary of FACS analysis of neural populations derived from control and Rest mutant ES cells with or without laminin treatment.

REST mutant	Cell type					
	*NSC*		*NPC*			*Neuron*
	Sox1^+^/Nestin^−^	Sox1^+^/Nestin^+^	Mash1^+^/Tau^−^	Mash1^+^/Tau^+^	Mash1^−^/Tau^+^	Map2^+^
**No laminin treatment**						
REST-100	20.7±0.8	77.4±1.3	47.2±2.7	41.7±3.6	1.2±0.2	83.1±2.7
REST/KD-50	20.9±1.2	76.2±1.6	44.5±2.6	36.4±1.8	1.6±0.5	81.3±1.0
REST-null	45.1±1.1**	52.9±2.1**	25.9±3.3*	24.4±1.3*	1.3±0.1	64.4±1.0**
REST-null+MTV	41.1±0.5**	54.5±1.9**	29.8±2.1*	22.7±1.4*	1.4±0.2	59.2±6.0**
REST-null+REST	33.9±1.1** ¥	62.9±1.4* #	39.1±1.6#	31.5±2.1#	1.1±0.1	77.6±0.3¥
**Laminin treatment**						
REST-100	14.6±1.3	78.8±3.1	38.3±2.1	31.8±2.4	0.5±0.1	74.8±0.3
REST/KD-50	13.8±1.6	77.1±2.1	40.8±2.3	28.5±2.6	0.4±0.1	74.1±0.4
REST-null	18.7±1.4	74.9±2.5	37.8±1.9	31.9±2.2	0.4±0.1	76.2±2.8
REST-null+MTV	19.1±1.5	75.2±2.3	37.1±2.2	29.5±2.1	0.5±0.1	75.3±1.8
REST-null+REST	17.9±1.1	78.4±1.8	35.9±2.4	31.4±1.8	0.6±0.2	75.9±2.1

Data are represented as mean±SEM.

*P<0.05 and **P<0.01, significantly different from REST-100 and REST/KD-50.

^#^ P<0.05 and ^¥^P<0.01, significantly different from REST-null and REST-null+MTV.

### Rest ablation impedes neuronal differentiation

Since Rest is known to be a repressor of neuronal gene expression and has been implicated in neurogenesis, we proceeded to examine the role of Rest in the generation of neurons from NSCs by assessing the expression of early and late neuronal markers (Fig. 1A). Similar to our studies on the generation of NSCs and NPCs, we noticed no significant difference in either the levels of gene expression, or in the number of cells expressing these neuronal markers in cells derived from REST/KD-50 ES cells compared with the control ES cells (Fig. 2F–2H; Table 1; Figs. S4B–S4C and S5B–S5C). However, REST-null ES cells showed a significant impairment in neuronal differentiation, as evidenced by a reduction in *Tubb3* expressing early neurons (P<0.01) (Fig. 2F), and a reduced population of Tau^+^ neurons (25%) compared to the control cells (42%) (P<0.05) (Table 1; Fig. S4B and S4D). Furthermore, we did not detect TujI^+^ (Tubb3) immunoreactive cells in differentiated REST-null cells at any earlier stage (data not shown), suggesting that Rest abrogation did not result in premature neuronal differentiation. As differentiation proceeded, the number of Map2^+^ mature neurons was significantly (P<0.01) reduced in REST-null cells (64%) compared with control cells (83%) (Table 1; Fig. S5B and S5D). Expression of *Scn2a* and *L1cam* (Fig. 2G–2H) both showed parallel changes. Intriguingly, even though *Tubb3*, *Scn2a* and *L1cam* are all Rest target genes, their expression were not de-repressed in REST-null ES cells during early ES cell differentiation (Fig. 2F–2H), a view consistent with the absence of any effect of acute Rest abrogation on expression of these genes in ES cells (unpublished observations). In conclusion, reduction of Rest levels by 50% has no effect on neurogenesis in terms of the time course, sequence or production of neurons. In contrast, the absence of Rest impedes neurogenesis.

### Constitutive expression of Rest rescues REST-null phenotypes

The REST-null ES cells were generated by a combination of gene targeting and RNAi knockdown. To confirm that their phenotype was not due to an off-target event, we constitutively expressed Rest using pMT-NRSF to create REST-null+REST, which raised *Rest* levels to 8% of wild-type levels. Transfection of empty pMT vector was used to generate REST-null+MTV that served as a control (Fig. S1C–S1D). We then assessed the capacity of REST-null+REST ES cells to differentiate into NSCs, NPCs and neurons. REST-null+REST ES cells generated NSCs expressing *Pax6* and *Msi1* at a similar level to that of REST-100 ES cells while *Nestin* was rescued to 80% of REST-100 levels. As predicted, REST-null+MTV cells showed a similar phenotype to the REST-null mutant (Fig. S6A–S6C). In support of these findings, the number of Nestin^+^ NS cells derived from REST-null+REST ES cells significantly increased to 63% (Table 1 and Fig. S3E–S3F). Moreover, constitutively expressing Rest rescued the capacity of REST-null mutant ES cells to produce NPCs, and neurons (Table 1, Figs. S4E–S4F and S5E–S5F). Our findings suggested that the phenotypic effects of REST-null ES cells are not due to off-target effects. Furthermore, our results indicate that very low levels of Rest are both sufficient and necessary for normal generation and maturation of NSCs.

### Rest ablation results in defects in adhesion, cell proliferation and survival

**Figure 3:**
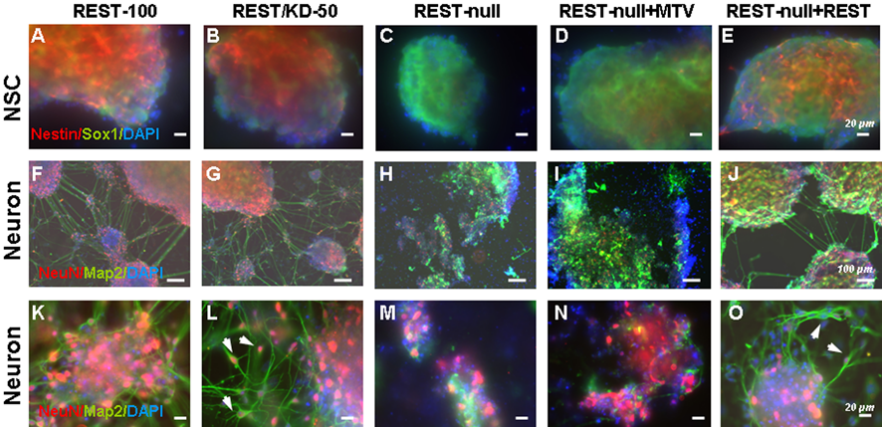
Phenotypic effects of Rest ablation on neural differentiation of ES cells. (A–E) NSCs were derived from the control (REST-100) and 4 Rest mutant ES cells, and identified by Sox1 (green) and Nestin (red) after 4 days of differentiation. Nuclei were stained with DAPI (blue). Few, if any, NSCs generated from REST-null ES cells (C and D) expressed Nestin, but showed positive immunostaining with Sox1. NSCs generated from control (REST-100) and REST/KD-50 ES cells showed much higher levels of Nestin immunostaining in Sox1^+^ cells (A–B). Constitutive expression of Rest (REST-null+REST) rescues, at least partially, this phenotype with many Sox1^+^ cells now expressing Nestin (E). (F–O) Neurons derived from the control and 4 Rest mutant ES cells were identified using NeuN (red) and Map2 (green) after 14 days of differentiation. Neurons generated from REST-100 and REST/KD-50 ES cells showed elaborate neurite outgrowth, fasciculation between aggregates/colonies (F, G and in higher power K, L) and migration of NeuN+ cells (see arrows in L). Conversely, neurons derived from REST-null and REST-null+MTV exhibited fragmented neuronal colonies that lacked elaborate processes and migration (H, I and in higher power M, N). Constitutively expressing Rest (REST-null+REST) attenuated the phenotypic effects of Rest ablation: neurite outgrowth, neurite fasciculation (J, O) and neuronal migration was observed (arrows in O). Scale bars: 20 µm (A to E and K to O) and 100 µm (F to J).

During investigation of the effects of Rest ablation on NSC and neuronal development, we observed that REST-null ES cells exhibited more severe phenotypes when plated on a glass surface than when plated on a plastic surface, whereas REST-100 and REST/KD-50 showed no discernible difference on either surface. On glass, REST-null cells showed defective adhesion and produced very few or no Nestin^+^ NSCs but did produce normal levels of Sox1^+^ NSCs as compared to those derived from REST-100 and REST/KD-50 ES cells (Fig. 3A–3C). This phenotype was much less pronounced when REST-null ES cells were plated on a plastic surface where a greater number of Nestin^+^ NSCs were seen (50%; Table 1). Intriguingly, NeuN^+^ and/or Map2^+^ neurons showed marked phenotypic defects on the glass surface, characterised by an absence of migration, and poor elaboration of processes and fasciculation among neuronal colonies as compared to REST-100 and REST/KD-50 ES cells (Fig. 3F–3H and 3K–3M). All phenotypes on the glass surface were rescued, at least in part, by raising *Rest* levels to 8% in REST-null+REST ES cells (Fig. 3D–3E; 3I–3J and 3N–3O). Interestingly, the phenotypic defects on the glass surface were also been seen in NSCs and neurons derived from *Rest−/−* ES cells [6] (Fig. S7C–S7K), although in the latter case the Rest mutant retains alternatively spliced isoforms that may not be functional (Fig. S7A–S7B).

**Figure 4:**
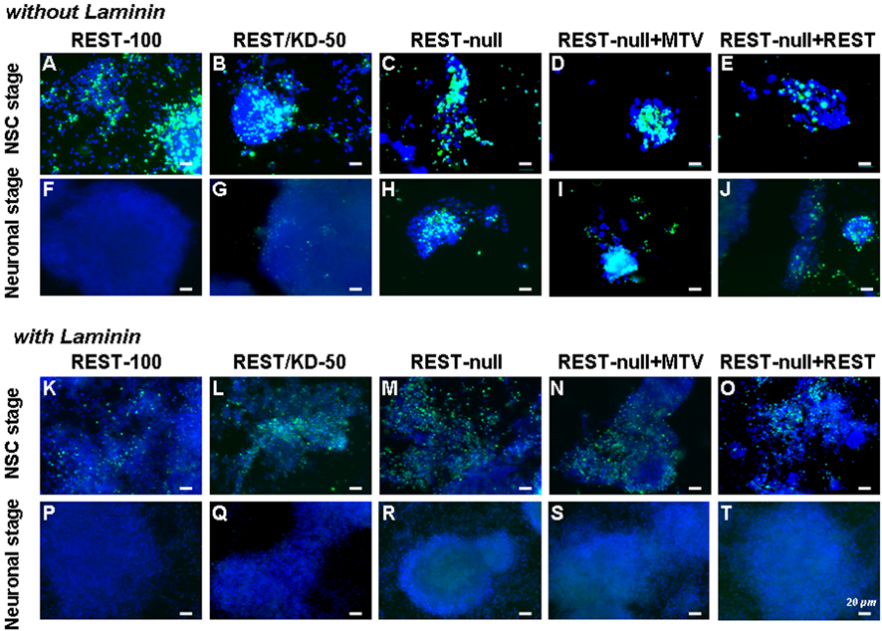
Laminins attenuate cell apoptosis caused by Rest ablation during neural differentiation of ES cells. The control REST-100 and 4 Rest mutant ES cells were either plated on gelatinised glass coverslips (A–J) or on glass coverslips coated with laminins (K–T) and subjected to standard neural differentiation. Cultures were fixed after 4 days of differentiation (A–E and K–O), the peak of NSC generation or fixed after 14 days of differentiation (F–J and P–T), when neurons were prevalent, and cell death in the cultures was assessed using TUNEL staining (green). In all cases cell nuclei were identified by DAPI staining (blue). As expected, cell death was observed when NSCs were generated in all groups (A–E). However, a marked reduction in cell death was observed in all cases when ES cells were plated and differentiated on laminin substrates (compare A–E with K–O). After 14 days of differentiation on gelatinised glass coverslips, markedly more cell death was observed in REST-null than in REST-100 and REST/KD-50 (compare H with F and G). A similar level of death was observed in REST-null+MTV (I). REST-null+REST exhibited less cell apoptosis (J). Laminin-treatment reduced apoptosis in all Rest mutants (compare H–J with R–T). Scale bar: 20 µm.

Either on plastic or glass surfaces, we found that REST-null ES cells generated fewer cells during neural differentiation than those from REST-100 and REST/KD-50 ES cells. Accordingly, we examined proliferation and cell death during the course of neurogenesis. Using BrdU incorporation, no differences were observed in the proliferation of either Sox1^+^/Nestin^−^ early NSCs derived from control and all Rest mutants or in Sox1^+^/Nestin^+^ late NSCs derived from the control, REST/KD-50 and REST-null+REST ES cells. However, it was difficult to evaluate the Sox1^+^/Nestin^+^ NSCs from REST-null and REST-null+MTV ES mutants, because of their low number and the ease with which they were lost from coverslips during the staining process. We then used TUNEL staining to assess the degree of apoptosis at 4 and 14-days of differentiation, i.e. at the peaks of NSC and neuron generation respectively. The ES monolayer culture system used in this study does not employ mitogens to induce neural differentiation [19], and accordingly, significant cell death occurs during NSC formation, especially during days 3 through 6 of differentiation, but thereafter this becomes much less marked as neuronal differentiation proceeds. At the NSC stage, cell death was equally prevalent in all samples derived from either control cells or Rest mutants (Fig. 4A–4E). However, at the neuronal stage, there was markedly more cell death in REST-null cells than those in the control and REST/KD-50 cells (Fig. 4F–4H), which is correlated with the ailing look of neurons derived from REST-null ES cells (Fig. 3H and 3I). Our results indicate that Rest ablation did not impair the proliferation of Sox1^+^/Nestin^−^ NSCs; however it is difficult to assess the proliferation of Sox1^+^/Nestin^+^ NSCs, due to the paucity of Nestin^+^ NSCs derived from REST-null ES cells. Moreover, *Rest* levels had to be reduced by more than 92% before an increase in cell death at the neuronal stage was observed.

### Laminins rescue the phenotype of the REST-null mutant

**Figure 5:**
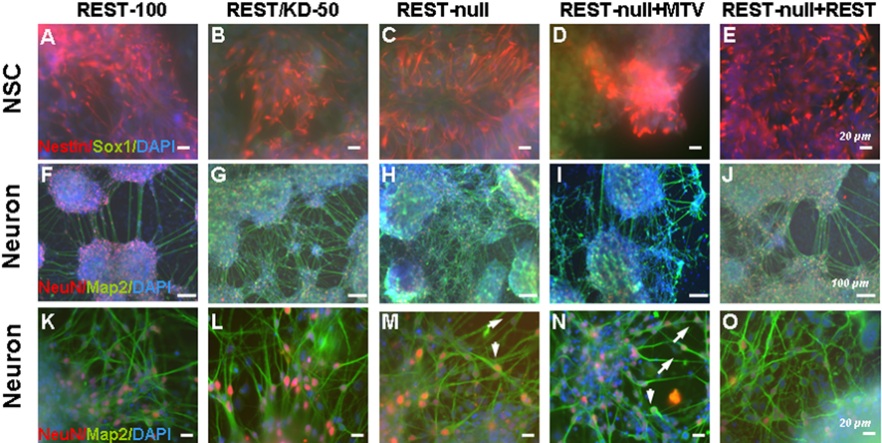
Laminins rescue the phenotypic effects of Rest ablation on the neural differentiation of ES cells. Control (REST-100) and 4 Rest mutants ES cells were grown under neural-differentiating conditions on laminin coated glass coverslips. After 4 days of differentiation some cultures were fixed and labelled with the NSC markers Nestin (red) and Sox1 (green) (A–E). The results show that laminins restored the ability to generate Nestin^+^ cells in Rest mutants (C–E, compared with Fig. 3C–3E). Sister cultures were allowed to differentiate for 14 days to analyse neuronal differentiation using NeuN (red) and Map2 (green) (F–J and in higher power K–O). Laminins rescued the neuronal phenotypes derived from REST-null ES cells, which included elaboration processes, neurite outgrowth and migration (see arrows in M and N) similar to that seen in neurons derived from control ES cells. Nuclei were stained with DAPI (blue) in all. Scale bars: 20 µm (A to E and K to O) and 100 µm (F to J).

REST-null defects in cell adhesion may be the cause of its phenotypic effects on Nestin^+^ NSC production and neuronal differentiation, because cell adhesion defects caused aberrant NSC and neuron development [20]–[21]. Thus, we further examined the causes of defective adhesion seen in the REST-null mutant. We and others have previously reported that several Rest target genes encode cell adhesion molecules or components of the extracellular matrix (ECM), particularly laminin subunits [7], [11]. Accordingly, we considered the notion that dysregulation of the ECM by Rest ablation might be responsible for this aspect of the phenotype. To test this hypothesis, we examined whether we could rescue the adhesion defect and any other phenotypic effects caused by Rest ablation by pre-treatment with ECM components. We plated ES cells from all groups on to either plastic or glass pre-treated with EHS laminins (which contain predominantly Laminin 1 (α1β1γ1)) [22] and subsequently subjected them to neural differentiation. On the glass surface pre-treated with laminins, but not those pre-treated with gelatin, the Rest mutant ES cells behaved like control ES cells, both of which adhered firmly and proliferated well. After 4-days of differentiation (NSC stage), cells from all groups plated onto glass surfaces pre-treated with laminins showed greater survival and significantly less apoptosis than those on untreated surfaces (Fig. 4K–4O). There was no significant difference in cell growth among the treated groups (the control and mutants). The surviving cells were highly proliferative as adjudged by BrdU incorporation (data not shown). Laminin pre-treatment had an even more profound effect after 14-days of differentiation (neuronal stage). No, or very few, apoptotic cells were detected in either the laminin-treated or control cells without laminin-treatment (Figs. 4F and 4P–4T), whereas significant cell death was found in the Rest mutants in the absence of laminin (Fig. 4H–4J). These data suggested that laminins prevented cell death in the REST-null cells. Intriguingly, the laminin rescue also extended to the specification of NSCs. We reported above that the REST-null ES cells were able to generate early Sox1^+^/Nestin^−^ NSCs but were unable to produce normal numbers of late Sox1^+^/Nestin^+^ NSCs when they were differentiated on a gelatinised glass surface in the absence of laminins (Fig. 3C–3D). This deficiency was rescued by laminin-treatment. Under these conditions, REST-null mutants, like control ES cells, generated equivalent numbers of both early NSCs (Sox^+^/Nestin^−^) and late NSCs (Sox^+^/Nestin^+^) (Fig. 5A–5E; Table 1). Furthermore, laminins rescued the production of NPCs and neurons from the REST-null mutant (Table 1). Neurons derived from REST-null ES cells showed widespread aggregation with extensive fasciculation (Fig. 5H–5I and 5M–5N) compared with those produced on glass in the absence of laminins (Fig. 3H–3I and 3M–3N).

To ensure that the rescue described above was not simply due to the enhanced adhesive properties of laminin, we investigated the ability of fibronectin (a different ECM component with similar adhesive properties to laminin) or poly–D-lysine (a commonly used non-biological substrate for neural cells) to mimic this response. Fibronectin was far less effective in rescuing the generation of Sox^+^/Nestin^+^ cells (late NSCs) from REST-null ES cells than laminin. Some Sox^+^/Nestin^+^ NSCs did develop when grown on fibronectin, however, the number observed was dramatically fewer when compared to that observed when REST-null cells were differentiated on laminins or when REST-100 cells were differentiated (Compare Fig. S8A with Fig. 5A and 5C). Some neurons were observed when REST-null cells were differentiated on fibronectin but again in far fewer numbers than seen after rescue with laminin or after differentiation of control cells (Fig. S8B). Phenotypically, neurons that developed on fibronectin had elongated process, suggesting that fibronectin was able to rescue the neuronal morphology of REST-null derived neurons, at least in part (Fig. S8B). The phenotype observed when REST-null ES cells were differentiated on poly-D-lysine was similar to that observed with gelatine; few Sox^+^/Nestin^+^ NSCs developed and all neurons observed looked ailing, being rounded and devoid of processes (compare Fig. S8A with Fig. 3C and Fig. S8B with Fig. 3H and 3M). Collectively, our results indicate that laminins rescued the adhesion defects seen during differentiation of REST-null ES cells and concomitantly rescued their ability to differentiate into Nestin^+^ NSCs, NPCs and neurons. This rescue cannot solely be attributed to the adhesive properties of laminin but must involve laminin-directed signalling since fibronectin has similar adhesive properties yet was dramatically less effective at rescuing both the NSC and neuronal differentiation.

### Impairment in laminins is caused by Rest ablation

**Figure 6:**
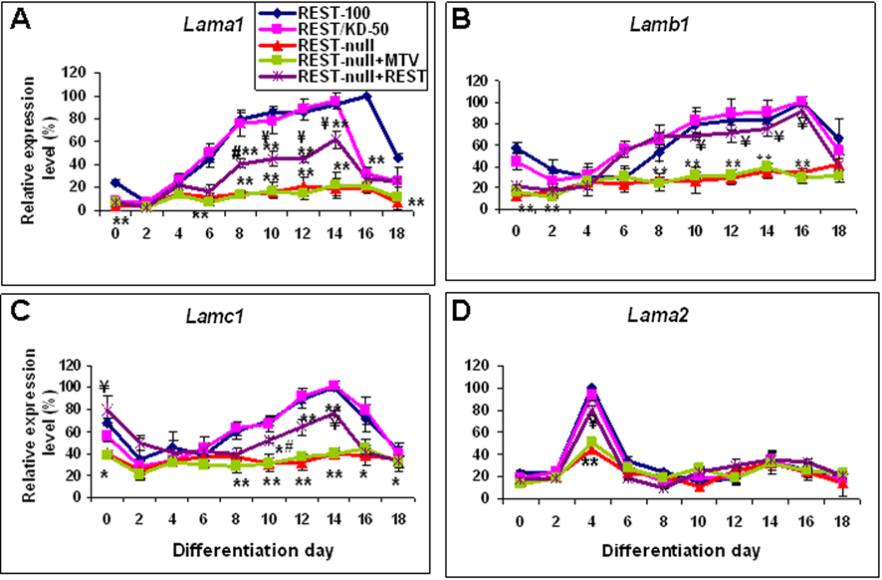
The effects of Rest ablation on the gene expression of laminin subunits during neural differentiation. The expression patterns of *Lama1* (A), *Lamb1* (B), *Lamc1* (C) and *Lama2* (D) were analysed throughout ES cell-derived neural differentiation by real time-PCR. Data are represented as mean±SEM. *P<0.05 and **P<0.01, significantly different from REST-100 and REST/KD-50. ^#^P<0.05 and ^¥^P<0.01, significantly different from REST-null and REST-null+MTV.

The laminin rescue of the REST-null phenotype indicated that laminins are downstream effectors of Rest. To test this idea, we determined whether the expression of laminins during neurogenesis was impaired by Rest ablation. Laminins have over 15 isoforms, each consisting of a combination of α, β, and γ subunits. In this experiment, we only focused on the effect of Rest deficiency on the expression of the genes encoding α_1_ (*Lama1*), β_1_ (*Lamb1*), γ_1_ (*Lamc1*) and α_2_ (*Lama2*) subunits since the EHS laminins used in the rescue experiments are composed mainly of laminin 1 (α_1_β_1_γ_1_). Furthermore α_2_ is a known Rest target gene [11] and a major component of the basement membrane in the embryonic CNS that is known to be involved in NSC development [23]. Each of these 4 laminin subunits exhibited distinct expression patterns during neural differentiation of ES cells (Fig. 6). In REST-100 and REST/KD-50 cells, the expression pattern of *Lama1* closely followed the time course of NSC and neuron development. Expression of *Lamb1* and *Lamc1* both showed a very similar pattern with high expression levels in ES cells followed by an initial decline during NSC differentiation and a subsequent gradual increase as neuronal differentiation proceeded. These expression patterns of *Lama1*, *b1* and *c1* are similar to those reported in an earlier *in vitro* study [24]. Intriguingly, the expression pattern of *Lama2* correlated closely with the time course of NSC formation, peaking at 4-days of differentiation, at a similar developmental stage as expression of α_2_ *in vivo* [23]. In contrast, REST-null cells exhibited significantly (P<0.01) decreased expression levels of *Lama1* throughout neurogenesis (Fig. 6A). Remarkably, raising the *Rest* level to 8% (REST-null+REST) restored *Lama1* levels to 40–50% of those of the control, although the levels remained significantly (P<0.01) lower than those seen in control cells. A similar change was also seen in the expression patterns of *Lamb1*, *c1* and *a2*. Rest ablation reduced the levels of *Lamb1*, *c1* and *a2* to below 40% of the level seen in control cells throughout neurogenesis, while raising the *Rest* level to 8% restored, at least partially, the expression levels of *Lamb1* and *a2* (Fig. 6B–6D). Taken together, our results suggest that Rest ablation impairs expression of laminins 1 and 2 (α_2_β_1_γ_1_) during neurogenesis, which leads to defects in cell adhesion, expansion and fasciculation.

## Discussion

Development of the vertebrate nervous system is orchestrated by transcriptional programs executed by both transcriptional activators and repressors. Studies suggested that the transcription factor Rest acts as a master regulator to suppress premature differentiation of neuronal progenitors and secure orderly neuronal maturation. Despite a wealth of information on the mechanism of Rest action and on identification of over 2000 target genes, we know very little about the biological function of Rest in the developing and mature nervous system. In our efforts to delineate the function of Rest during neural development, we generated several Rest deficient ES cell mutants to elucidate the role of Rest in the transition from pluripotent ES cells to multipotent NSCs and subsequently to mature neurons. Here, we show that deletion of a single *Rest* allele has no discernible effect on either NSC formation or neurogenesis but severe depletion of *Rest* to levels less than 8% impedes NSC and neuron development, and further that these impairments are mediated by attenuation of laminin levels.

### Rest is dispensable for ES cell pluripotency

Recently, a study showed that knocking down Rest by 50% altered ES cell pluripotency and promoted ES cell differentiation into endoderm, mesoderm and ectoderm in ES medium [9]. However, our findings show that depletion of Rest levels by 50% to 100% does not change ES cell pluripotency as assessed by expression of pluripotent markers and by lineage competence. In this study, we have generated Rest mutant ES cells, which expressed Rest at 50%, 8% and 0% of the wild-type level. We found that these mutant ES cells did not significantly decrease the expression of *Oct4* and *Nanog*, a finding that is congruent with observations of *Rest+/−* and *Rest−/−* ES cells derived from Rest knockout mice in which gastrulation proceeds normally [6]. Moreover, we also found that two independently derived ES lines with only one allele of *Rest*, REST/KD-50 and *Rest+/−*, both behave like their wild-type counterparts, and are fully capable of normal *in vitro* neural differentiation. These findings are consistent with the findings that, *in vivo*, *Rest+/−* ES cells are pluripotent and are able to generate germ-line mice. Additionally, the mice derived on a *Rest+/−* background showed no discernable phenotypic changes as compared to their wild-type litter-mates [6]. These findings suggest that Rest haplodeficiency does not alter ES cell pluripotency.

### Rest ablation impedes NSC and neuron development

There are over 2000 RE1 sites in the human and murine genomes [12] and many RE1-bearing genes encode ion channels, neurotransmitters, growth factors and hormones, and factors involved in axonal guidance and vesicle trafficking, and molecules involved in maintenance of the cytoskeleton [5], [25]. The observation that many of these Rest target genes are expressed by postmitotic neurons, helped to foster the initial belief that the role of Rest was to prevent premature expression of neuron-specific genes in NPCs [26]–[27], [6]. Further, it has been proposed that down-regulation of Rest in NPCs is required for their subsequent differentiation [8]. However, we found that Rest ablation in ES cells and NSCs results neither in increased NSC production nor in precocious neuronal differentiation. Rather, Rest ablation significantly decreases the production of Nestin^+^, *Pax6-* or *Msi1*-expressing NSCs without affecting that of Sox1^+^ NSCs, suggesting that Nestin^+^ NSCs are derived from Sox1^+^ NSCs. This view is confirmed by previous studies showing that the expression of *Nestin* is regulated by co-operative action between Sox1-3 and class III POU transcription factors [28]. Our results indicate that the function of Rest is not restricted to regulating neuronal differentiation as previously conceived, but that Rest also takes part in upstream events regulating the generation of Nestin^+^ NSCs from Sox1^+^ NSCs. This regulation of early NSC development is echoed in a previous study that showed that disruption of Rest function in Xenopus disturbs ectoderm patterning and expands the neural plate [18].

Additionally, Rest ablation also reduces the production of Mash1^+^ (and *Mash1* ^+^) or *Ngn1^+^* NPCs. This may be secondary to the diminished NSC population since there are no detectable differences in proliferation or cell death during derivation of NSCs from either the control or REST-null ES cells. Our data show that the role of Rest in NSCs and NPCs is not simply to repress expression of neuronal target genes and to prevent precocious neuronal differentiation, but rather that Rest plays manifold roles at the level of both NSC and NPC generation.

We also find that the absence of Rest in ES cells and NSCs does not cause precocious neuronal differentiation. In fact, even direct Rest target genes, such as the neuronal markers *Tubb3*, *L1cam* and *Scn2A*, are not precociously expressed. This finding resonates strongly with the initial experiments on *Rest−/−* mice, which displayed embryonic lethality around E11, but importantly, showed no widespread precocious expression of Rest target genes. Only *Tubb3* was seen to be de-repressed in non-neuronal tissues but not in the developing CNS. Furthermore, constitutive expression of Rest in chick spinal cord causes axon pathfinding errors, but only two Rest target genes encoding *Tubb3* and *L1cam* were repressed [17]. Taken together, our findings suggest that the role of Rest in neuronal differentiation is not to simply regulate its neuronal target genes by a simple “on-off” switch. The regulation of Rest in these target genes may be compounded by other transcriptional repressors or co-repressors, such as has been reported for SMCX, a transcriptional repressor that co-regulates Rest neuronal target genes in X-linked mental retardation [29] and NFkappaB that synergistically interacts with Rest in the suppression of *TAC1* in non-neuronal cells [30].

Strikingly, there is also no evidence that the absence of Rest promotes or increases neuron number [31]–[32]. On the contrary, we find that Rest knockout decreases neuron production and causes severe defects in neuronal interaction (judged by malformation of neuronal processes and fascicles and neuronal migration). The decline in neuron production, in part, is attributed to cell death, because we found that Rest ablation increases neuronal death. These phenomena however can be rescued by increasing levels of *Rest* expression, indicating that Rest is a causal effector. Taken together, the role of Rest during neuronal development is not restricted simply to repress neuron-specific genes in NPCs prior to terminal neuronal differentiation.

### Laminin deficiency causes impairment in NSC and neuron development in REST-null ES cells

In this study, the most striking phenotype of Rest mutant ES cells is their defective adhesion, especially on a glass surface. The other phenotypes include impairment in the production of Nestin^+^ NSCs (albeit the mutants can generate a comparable amount of Sox1^+^ NSCs), and defects in neuronal migration and process formation. Additionally, Rest ablation results in greater cell death and a consequent reduction in the number of neurons. Interestingly, we also observed these phenotypes in ES cells derived from *Rest−/−* mice [6]. Our finding suggests these are *bona fide* phenotypes of Rest-null mutants and not due to the artefact of a single ES cell line used in this study. In previous studies, we and others identified several Rest target genes that are involved in cell-matrix adhesion and cell-cell interaction, such as *Lama1*, *Lama2*, *Arc*, *Cspg3*, *Unc5d*, *Adam23*, *Catnd2*, *Cdh4*, *L1-cam* and *Mmp24* [7], [11], [12]. This latter observation prompted us to consider the possibility that dysregulation of cell-matrix adhesion molecules may be responsible for Rest-knockout phenotypes.

Cell-matrix adhesion molecules mediate direct interactions of the cell with its extracellular environment by binding of cell surface molecules with components of ECM, and are crucial for cell migration, differentiation, organisation and embryogenesis [33]–[35]. In the developing central nervous system, the ECM is present in the basement membrane of the ventricular zone and is essential for ordered differentiation of neuronal subtypes in the cerebral cortex [36]. *In vitro*, the ECM has been shown to play an important role in lineage decision and cell type selection [21], [37]. The ECM mainly consists of laminins, fibronectin, type IV collagen, nidogen and heparan sulfate proteoglycans [38]–[39]. Laminins are a family of extracellular glycoproteins expressed throughout developing neural tissues and each laminin is composed of α, β, and γ polypeptide chains [40]. In this study, EHS laminins rescue the phenotypes of Rest mutant, by restoring their adhesion ability and their differentiation into Nestin^+^ NSCs, NPCs and mature neurons. Not only do laminins promote neuron migration, neurite outgrowth and elaboration, but they also prevent neuronal cell death caused by Rest ablation. These findings resonate with the known functions of laminins in the NS cell niche where they are required for proliferation, neuronal differentiation and survival, and neurite outgrowth [20], [23], [41]. Additionally, we found that although fibronectin exhibited similar adhesiveness to that of laminins, it nevertheless was markedly less effective than laminins in restoring the REST-null phenotype, indicating that specific ECM signalling rather than adhesion alone is the key player in REST-null phenotypes.

How does Rest impair the expression of laminins, which subsequently generate phenotypic effects in Rest mutants? We know that the genes encoding laminin subunits α1 and α2 are direct Rest target genes [11] and γ1 is an indirect target since it is regulated by miR-124, which is a direct Rest target gene [42]. During neural differentiation of ES cells, the expression pattern of *Lama1* showed an inverse relationship with that of *Rest*, suggesting Rest may act as a repressor in *Lama1* expression. However, Rest ablation in ES cells caused impairment in stage-dependent *Lama1* expression, indicating that a minimal amount of Rest is required to maintain *Lama1* expression. That this is the case can be seen from the rescue of *Lama1* expression by raising *Rest* levels to 8%. This phenomenon also applies to the *Lama2* expression. In fact, the expression of *laminin* subunits correlates with severity of phenotypic effects of Rest deficiency. In conclusion, our studies show that one of the roles of Rest in neural development is to regulate ECM components, which in turn are required for the transition from ES cell to neural lineage. Furthermore, we show that Rest is required at multiple stages during neural development from production of NSCs through to terminal neuronal differentiation. This contrasts with the contemporary idea that limits Rest to regulation of neuronal development by simply repressing neuron-specific target genes prior to loss of Rest expression during the final stages of terminal neuronal differentiation.

## Materials and Methods

### Generation of Rest mutant ES cells

To generate Rest mutant ES cells, firstly, we have constructed a conditional knockout targeting vector, REST/ck vector (Fig. S1A) to replace one allele of the *Rest* gene in HM1 ES cells (a gift from Dr. J. Mcwhir, the Roslin Institute) using homologous recombination. In general, HM1 ES cells were transfected with the REST/ck vector digested with Afl II/Bgl II using electroporation (800 V, Time constant 0.2 msec) and in G418 selection from the second day after transfection. Colonies were screened for targeted clones using RT-PCR with two primer pairs, primers 1/2 and primers 3/4 (Fig. S1A). Of 500 colonies, one clone was identified as a REST/ck-targeted clone, which was used to create the control ES cells (called REST-100) and Rest+/− ES cells (called REST/KD-50). REST-100 ES cells were generated by stably transfecting with the empty pSuper vector, whereas REST/KD-50 ES cells were created by transiently expressing *Cre* recombinase (a gift from Dr. Jeremy Brown) to delete one allele of *Rest*. The REST/KD-50 ES cells were then used to create REST-null ES cells by stably expressing a Rest shRNA (5′-GTGTAATCTACAATACCAT-3′) in the presence of puromycin (2 µg/ml) using a pSuper-Rest-shRNA vector [43].

The *Rest* levels expressed from REST-100, REST/KD-50 and REST-null ES cells were 100%, 50% and 1%, respectively as adjudged by quantitative Real-time PCR (Fig. S1B), and confirmed by Western blot analysis (Fig. S1D). In our Western blot analysis, the level of Rest in REST-100 (lane 1) is approximately double that seen in REST/KD-50 (lane 2) when normalised to levels of the house-keeping gene, Gapdh (lower panel) (Fig. S1D). Although REST-null ES cells produced 1% of wild type *Rest* mRNA levels, as detected by PCR analysis, Rest protein level was undetectable by Western blot analysis using a Rest antibody (Upstate; 07-579) at a 1∶1000 dilution. Therefore, we consider this to be a Rest-null mutant. For a rescue experiment, we constitutively expressed *Rest* in the REST-null ES cells with the pMT-NRSF vector (a gift from David Anderson, Caltech [27]) to produce REST-null+REST, which expresses 8% of the control *Rest* levels. Control cells were produced by transfection of REST-null ES cells with empty pMT vector (REST-null+MTV) (Fig. S1C–S1D). Although Rest in REST-null+REST ES cells was not detectable by Western blot analysis, it was detected by immunocytochemical analysis (data not shown). We also showed that *Rest* is effectively silenced throughout the neuronal differentiation process (Fig. S1B–S1C). Furthermore, in contrast to recent observations [9], we also found that Rest deficiency (50% to 100%) did not affect the pluripotency of ES cells as adjudged by the expression levels of *Oct4* and *Nanog* (Fig. S1E).

### Quantitative Real Time-PCR

Primer design and experimental details were carried out as described previously [7] and in Methods S1. Primers used in this study are shown in Table S1. All expression levels were normalised to cyclophillin levels and then as a percentage of the highest level of expression of the REST-100 clone. All data were performed in duplicate and repeated three times.

### Statistical Analysis

Statistical significance was determined using a two-tailed Student test. P values of <0.05 and <0.01 were considered statistically significant. All results are presented as mean±standard error of the mean (SEM) from experiments that have been repeated three times.

### Neuronal differentiation

Rest mutant and control ES cells were differentiated into NSCs, NPCs and then neurons using a monolayer culture in N2B27 medium [19]. For studying gene expression patterns and for cell type analysis, control and Rest mutant ES cells (600,000 cells/plate) were plated onto 10-cm Petri dishes coated with 0.1% gelatine in ES medium. The next day, the ES cell medium was replaced with N2B27 medium. Medium was changed every 2 days and differentiation continued for 18 days. For studying gene expression patterns, samples were collected at day 0 (ES cell stage, before differentiation) and 2, 4, 6, 8, 10, 12, 14, 16 and 18 days after differentiation. For cell type analysis using FACS, samples were collected separately from 4, 10 and 14 days of differentiation, which cells were plated out either on gelatine or laiminin-treated surface. In immunocytochemical analysis, ES clones were seeded onto gelatinised, polyornithine/fibronectin (10 µg/ml, Sigma), polyornithine/laminin (10 µg/ml, Sigma) or poly-D-lysine (60 µg/ml, Sigma) coated glass coverslips (VWR, 631-0150) in 24-well plates at a density of 1×10^4^/cm^2^ in ES cell medium. The next day, cells were subjected to the differentiation process in N2B27 medium. All experiments were repeated three times.

### Immunocytochemistry

ES cells, NSCs and neurons were fixed in 3% paraformaldehyde for 20 min at RT. Antibodies, Nestin (1∶500, MAB353, Chemicon) and Sox1 (1∶500, AB5934, Chemicon) were used to identify neural stem cells, whereas NeuN (1∶500, MAB377, Chemicon) and MAP2 (1∶400, ab10588-50, Abcam) were used for staining neurons. All primary antibody staining was carried out at 4°C, overnight. Samples were then stained with appropriate fluorescence-conjugated secondary antibodies at RT for 1 hr and examined under an Axiovision fluorescent microscope (Zeiss).

### Flow cytometry

Cells were collected 4, 10 and 14 days after differentiation for FACS analysis of NSCs, NPCs/early neurons and mature neurons, respectively. Cells were trypsinised to dissociate into single cells, fixed in 3% paraformaldehyde for 15 min, permeabilsed with 0.1% saponin (Invitrogen) for 30 min and then stained with Nestin/Sox1 (both 1∶250) or Mash1(Chemicon)/Tau (Chemicon) (both 1∶200) or Map2 (1∶300) in the presence of 0.1% saponin for 1 hr. All procedures were carried out at room temperature. Cells were subsequently stained with their corresponding secondary antibodies: Cy3-anti-mouse/FITC-anti-chicken (both 1∶300) for Nestin/Sox1, Cy3-anti-goat/FITC-anti-rabbit (both 1∶300) for Mash1/Tau and FITC-anti-chicken (1∶300) for Map2. The corresponding controls were stained only with secondary antibodies. Cells were acquired with a FACS LSR with CellQuest software (BD biosciences). Flow cytometry data were analysed using Summit v4.3 (Dako Colorado, Inc). All FACS analysis experiments were repeated three times.

### Cell proliferation and apoptosis

5-bromo-2-deoxyuridine (BrdU; 10 µM; Sigma) was added to ES cells grown on polyornithine/laminin (10 µg/ml) coated 13 mm glass coverslips. 16 hrs later, cultures were fixed with 2% paraformaldehyde for 15 min, followed by 95% methanol for 30 min at room temperature. After rinsing with PBS, coverslips were incubated with a biotin conjugated sheep anti-BrdU antibody (1∶250; Abcam) overnight at 4°C in PBS containing 0.1% Triton X-100 and 10% normal goat serum (Sigma). BrdU labelled cells were visualised using streptdavidin conjugated to Alexa Fluor 488 (1∶500; Invitrogen). After three washes with PBS, coverslips were mounted on slides using Fluoromount-G (Invitrogen) and analysed using an Axiovision fluorescent microscope (Zeiss). Apoptotic cell death was detected by terminal deoxyribonucleotidy transferase-mediated dUTP-digoxigenin nick end labelling (TUNEL) according to manufacturer's instructions (Promega).

## Supporting Information

Figure S1 The generation of Rest mutants. (A) The Rest conditional knockout targeting vector (REST/ck) (bottom) was designed according to *Rest* gene (top). The REST/ck vector was constructed by inserting a FRT-tk-neor-FRT-LoxP cassette at the Aat II site in intron 2 and a LoxP site at the Asc I site in the exon 1 of *Rest* gene. This design is to abrogate Rest production by snapping off exons 1 and 2, which encode the transcriptional start sites, the N terminal repression domain and 4 zinc fingers of Rest protein, in the presence of Cre recombinase. Primers shown were used to identify targeted clones. (B–C) *Rest* expression levels during ES cell-derived neural development from the control (REST-100), REST/KD-50, REST-null, REST-null+MTV and REST-null+REST. (Data are represented as mean±SEM.) (D) Western blot analysis in the control and Rest mutants. Lane 1: REST-100; 2: REST/KD-50; 3: REST-null; 4: REST-null+MTV and 5: REST-null+REST. Gapdh as an internal control. (E) The pluripotency of embryonic stem (ES) cells was not altered in Rest mutants judged by the expression of ES cell markers, *Oct4* and *Nanog*. (Data are represented as mean±SEM.)
(0.15 MB TIF)

Figure S2 Gene expression patterns of stage-specific markers during 46C ES-derived neural differentiation. ES cell differentiation into neurons recapitulates neurogenesis in vivo down-regulation of ES cell pluripotent marker *Oct4* and *Rest* (A) to the sequential development, firstly neural stem cells (*Nestin* and *Pax6*) (B), then neural progenitor cells (*Mash1* and *Ngn1*) (C) and to neurons (*Syn1*, *L1cam* and *Tubb3*) (D). This experiment corroborated the findings in HM1 ES cells.
(0.07 MB TIF)

Figure S3 FACS analysis of Sox1^+^ and/or Nestin^+^ NSCs derived from control and Rest mutant ES cells. (A) Background control showing control (REST-100) cells co-stained with the secondary antibodies (Cyc3-anti-mouse/FITC-anti-chicken) used to visualise the anti-Nestin and anti-Sox1 antibodies respectively. (B–F) Nestin and Sox1 expression on NSCs generated from control and Rest mutant ES cells. Cells in the R5, R2 and R3 areas are classified as Sox1^+^, Nestin^+^ and Sox1^+^/Nestin^+^ respectively. These NSC populations are summarised in Table 1.
(0.25 MB TIF)

Figure S4 FACS analysis of Mash1^+^ and/or Tau^+^ NPCs/early neurons from control and Rest mutants. (A) Background control showing control (REST-100) cells co-stained with the secondary antibodies (Cyc3-anti-goat/FITC-anti-rabbit) used to visualise to the anti-Mash1 and anti-Tau antibodies respectively. (B–F) Mash1 and Tau expression on NPCs/early neurons generated from control and Rest mutant ES cells. Cell in the R5, R2 and R3 areas are classified as Tau^+^, Mash1^+^ and Tau^+^/Mash1^+^ respectively. These populations of NPCs/early neurons are summarised in Table 1.
(0.22 MB TIF)

Figure S5 FACS analysis of Map2^+^ neurons from control and Rest mutant ES cells. (A) Background control showing control (REST-100) cells stained with the secondary antibody (FITC-anti-chicken) used to visualise to the anti-Mash1 antibody. (B–F) Map2 expression on neurons generated from control and Rest mutant ES cells. Cells in the R5 area are classified as Map2^+^ mature neurons. This population of mature neurons is summarised in Table I.
(0.21 MB TIF)

Figure S6 Constitutively expressing *Rest* in REST-null ES cells rescues their differentiation defects. By constitutively expressing *Rest* in REST-null cells (REST-null+REST) using pMT-NRSF, we raised *Rest* levels to 8% of wild-type levels. We compared the ability of REST-null+REST ES cells to generate NSCs, NPCs and neurons with that of the REST-null ES cells transfected with empty vector (REST-null+MTV) by real time-PCR analysing the expression of stage-specific differentiation markers: *Pax6*, *Msi1* and *Nestin* to detect neural stem cells (NSCs) (A–C); *Ngn1* and *Mash1* to detect neural progenitor cells (NPCs) (D, E); *Tubb3* to detect young neurons (F) and *Scn2a* and *L1cam* to detect mature neurons (G–H). Raising *Rest* expression level to 8% restored the gene expression levels of stage-specific markers to those observed in the control ES cells. Data are represented as mean±SEM. *P<0.05 and **P<0.01, significantly different from REST-null+REST.
(0.09 MB TIF)

Figure S7 Confirmation of REST-null phenotypes in the ES cells derived from Rest knockout mice (Chen et al., 1998). (A) Rest protein expression analysed by Western blot. An alternatively spliced Rest isoform, which has a deletion in the entire exon 2 encoding the N-terminus of Rest, can be seen in ES cells derived from *Rest+/−* and *Rest−/−* mice. Gapdh was used as an internal control. (B) The expression levels of *Rest* in wild-type, *Rest+/−* and *Rest−/−* ES cells quantified by real-time PCR using primer sets designed against the N-ternimus and the C-terminus of *Rest* gene. *Rest* is expressed in *Rest−/−* ES at the wild-type level when primers against the C-terminus of *Rest* were used, indicating the existence of an alternatively spliced isoform which corroborated the result analysed by Western blot analysis (A). (C–E) The neural stem cells (NSCs) derived from wild-type, *Rest+/−* and *Rest−/−* ES cells were identified by Sox1 (green) and Nestin (red) from a 4-day differentiation. Nuclei were stained with DAPI (blue). The NSCs from *Rest−/−* ES cells show less Nestin expression in Sox1^+^ cells as compared to those from wild-type and *Rest+/−* ES cells, which is resemble to the finding in REST-null mutants (Fig. 3C and 3D). (F–H) Low power and (I–K) high power images of neurons derived from the wild-type, *Rest+/−* and *Rest−/−* ES cells. Neurons were identified by NeuN (red) and Map2 (green) after 14 days of differentiation. Nuclei were counterstained with DAPI (blue). The neurons generated from wild-type and *Rest+/−* ES cells show similar phenotypes to those derived from REST-100 and REST/KD-50 ES cells (Fig. 3F–G and K–L), whereas the neurons from *Rest−/−* to those from REST-null ES cells, which are devoid elaborated processes and migration (Fig. 3H–I and M–N).
(0.66 MB TIF)

Figure S8 The effect of fibronectin and ploy-D-lysine on REST-null phenotypes. Control (REST-100) and REST-null ES cells were plated onto coverslips coated either with poly-D-lysine or fibronectin. The next day, cells were driven along a neural differentiation pathway in B2N27 medium. After 4 days of differentiation cells were fixed and analysed for the presence of neural stem cells by staining with Sox1 (green) and Nestin (red) (A) and the presence of neurons by staining with Map2 (green) and NeuN (red) after 14 days of differentiation (B). Nuclei were stained with DAPI (blue) (scale bar: 25 µm). All images were captured using a Zeiss fluorescence microscope equipped with an ApoTOME.
(0.73 MB TIF)

Methods S1 Supplemental data
(0.03 MB DOC)
