# Supplementary figures and images for: Rest-Mediated Regulation of Extracellular Matrix Is Crucial for Neural Development
Source: PLoS One. 2008 Nov 6;3(11):e3656. doi: 10.1371/journal.pone.0003656 (PMC2573962; doi:10.1371/journal.pone.0003656)

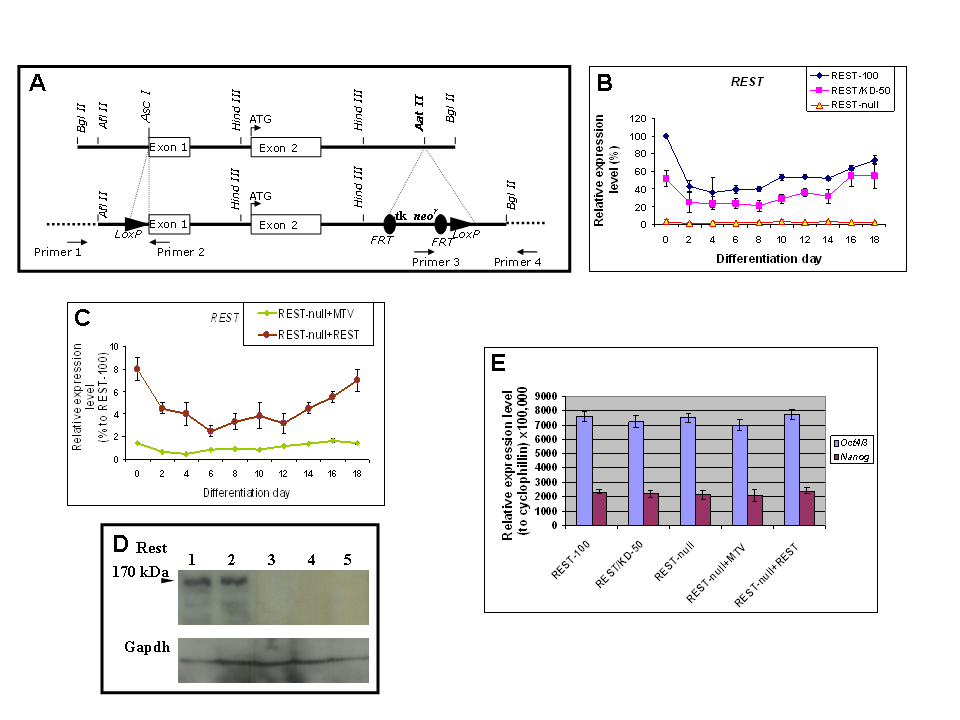

Supplement: Figure S1 — The generation of Rest mutants. (A) The Rest conditional knockout targeting vector (REST/ck) (bottom) was designed according to Rest gene (top). The REST/ck vector was constructed by inserting a FRT-tk-neor-FRT-LoxP cassette at the Aat II site in intron 2 and a LoxP site at the Asc I site in the exon 1 of Rest gene. This design is to abrogate Rest production by snapping off exons 1 and 2, which encode the transcriptional start sites, the N terminal repression domain and 4 zinc fingers of Rest protein, in the presence of Cre recombinase. Primers shown were used to identify targeted clones. (B–C) Rest expression levels during ES cell-derived neural development from the control (REST-100), REST/KD-50, REST-null, REST-null+MTV and REST-null+REST. (Data are represented as mean±SEM.) (D) Western blot analysis in the control and Rest mutants. Lane 1: REST-100; 2: REST/KD-50; 3: REST-null; 4: REST-null+MTV and 5: REST-null+REST. Gapdh as an internal control. (E) The pluripotency of embryonic stem (ES) cells was not altered in Rest mutants judged by the expression of ES cell markers, Oct4 and Nanog. (Data are represented as mean±SEM.) (0.15 MB TIF) [file pone.0003656.s001.tif]

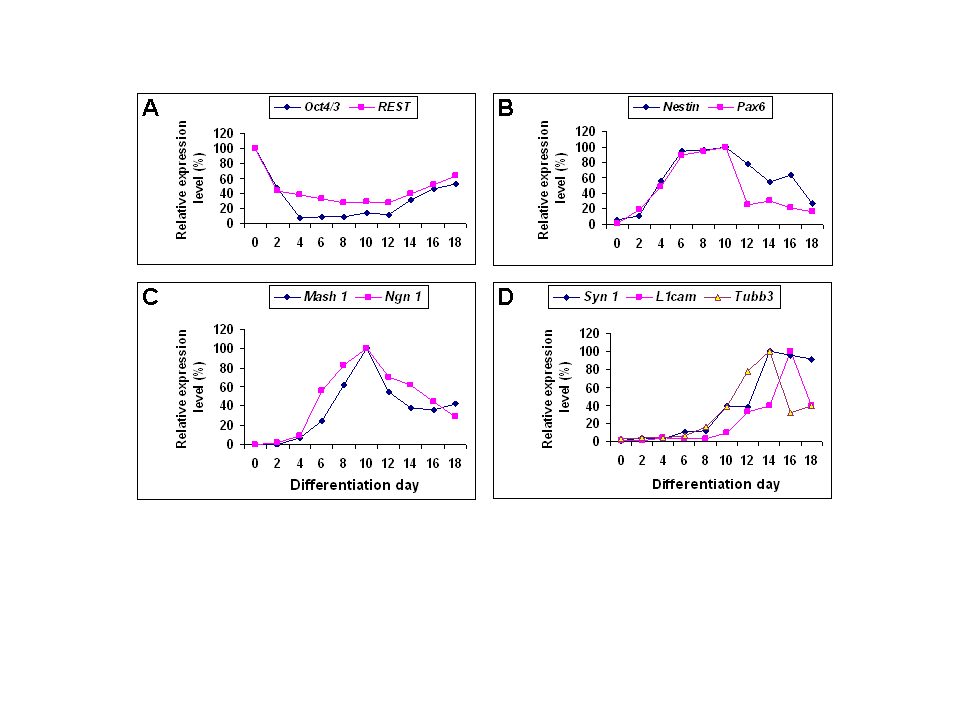

Supplement: Figure S2 — Gene expression patterns of stage-specific markers during 46C ES-derived neural differentiation. ES cell differentiation into neurons recapitulates neurogenesis in vivo down-regulation of ES cell pluripotent marker Oct4 and Rest (A) to the sequential development, firstly neural stem cells (Nestin and Pax6) (B), then neural progenitor cells (Mash1 and Ngn1) (C) and to neurons (Syn1, L1cam and Tubb3) (D). This experiment corroborated the findings in HM1 ES cells. (0.07 MB TIF) [file pone.0003656.s002.tif]

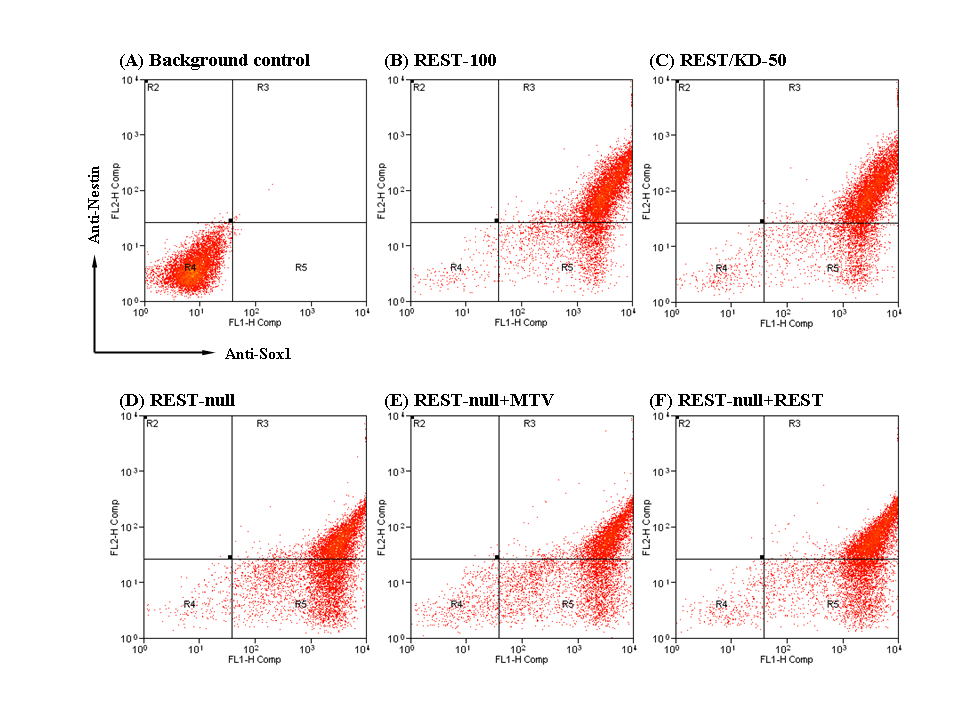

Supplement: Figure S3 — FACS analysis of Sox1+ and/or Nestin+ NSCs derived from control and Rest mutant ES cells. (A) Background control showing control (REST-100) cells co-stained with the secondary antibodies (Cyc3-anti-mouse/FITC-anti-chicken) used to visualise the anti-Nestin and anti-Sox1 antibodies respectively. (B–F) Nestin and Sox1 expression on NSCs generated from control and Rest mutant ES cells. Cells in the R5, R2 and R3 areas are classified as Sox1+, Nestin+ and Sox1+/Nestin+ respectively. These NSC populations are summarised in Table 1. (0.25 MB TIF) [file pone.0003656.s003.tif]

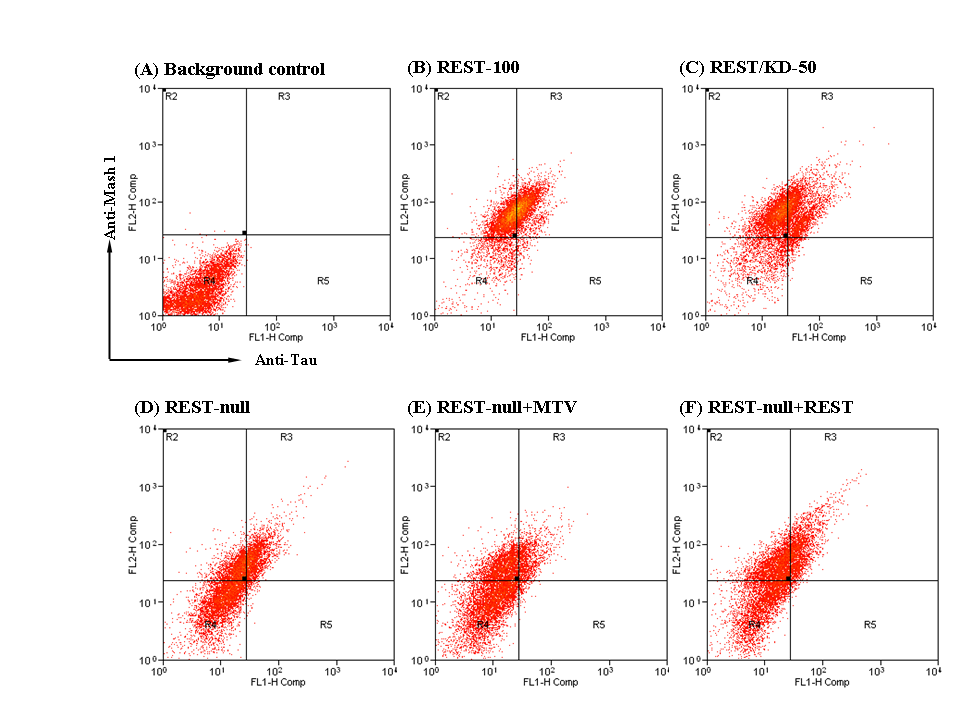

Supplement: Figure S4 — FACS analysis of Mash1+ and/or Tau+ NPCs/early neurons from control and Rest mutants. (A) Background control showing control (REST-100) cells co-stained with the secondary antibodies (Cyc3-anti-goat/FITC-anti-rabbit) used to visualise to the anti-Mash1 and anti-Tau antibodies respectively. (B–F) Mash1 and Tau expression on NPCs/early neurons generated from control and Rest mutant ES cells. Cell in the R5, R2 and R3 areas are classified as Tau+, Mash1+ and Tau+/Mash1+ respectively. These populations of NPCs/early neurons are summarised in Table 1. (0.22 MB TIF) [file pone.0003656.s004.tif]

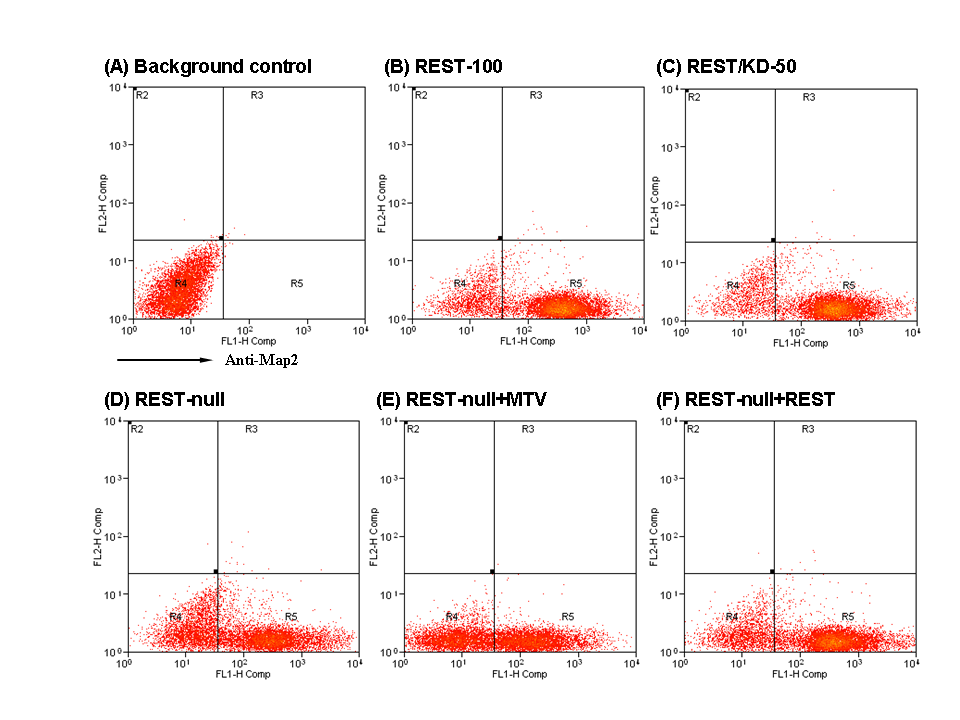

Supplement: Figure S5 — FACS analysis of Map2+ neurons from control and Rest mutant ES cells. (A) Background control showing control (REST-100) cells stained with the secondary antibody (FITC-anti-chicken) used to visualise to the anti-Mash1 antibody. (B–F) Map2 expression on neurons generated from control and Rest mutant ES cells. Cells in the R5 area are classified as Map2+ mature neurons. This population of mature neurons is summarised in Table I. (0.21 MB TIF) [file pone.0003656.s005.tif]

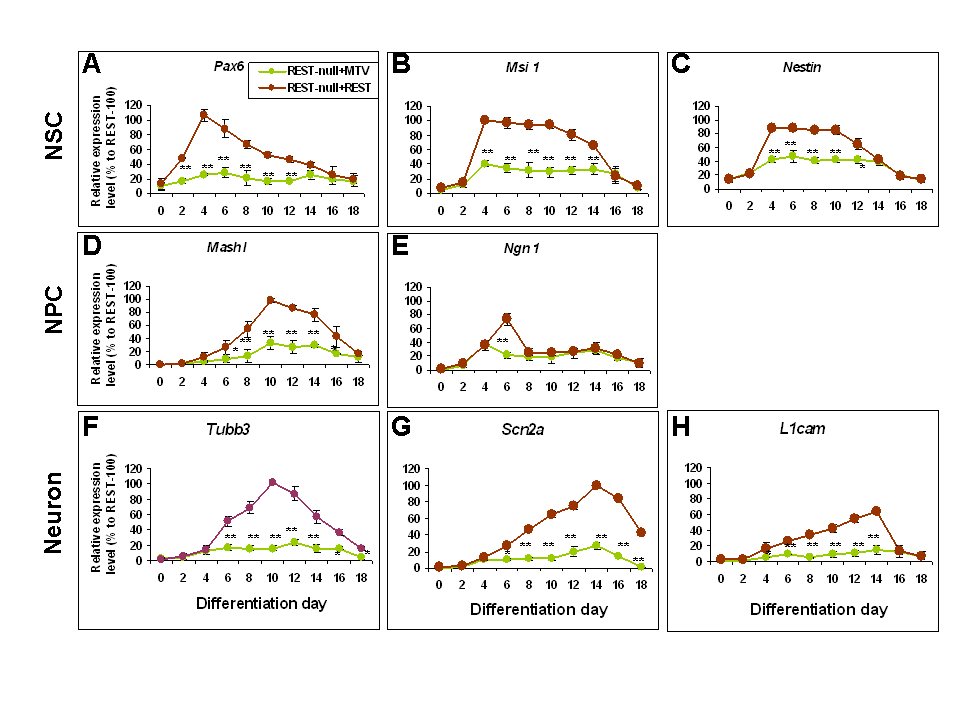

Supplement: Figure S6 — Constitutively expressing Rest in REST-null ES cells rescues their differentiation defects. By constitutively expressing Rest in REST-null cells (REST-null+REST) using pMT-NRSF, we raised Rest levels to 8% of wild-type levels. We compared the ability of REST-null+REST ES cells to generate NSCs, NPCs and neurons with that of the REST-null ES cells transfected with empty vector (REST-null+MTV) by real time-PCR analysing the expression of stage-specific differentiation markers: Pax6, Msi1 and Nestin to detect neural stem cells (NSCs) (A–C); Ngn1 and Mash1 to detect neural progenitor cells (NPCs) (D, E); Tubb3 to detect young neurons (F) and Scn2a and L1cam to detect mature neurons (G–H). Raising Rest expression level to 8% restored the gene expression levels of stage-specific markers to those observed in the control ES cells. Data are represented as mean±SEM. *P<0.05 and **P<0.01, significantly different from REST-null+REST. (0.09 MB TIF) [file pone.0003656.s006.tif]

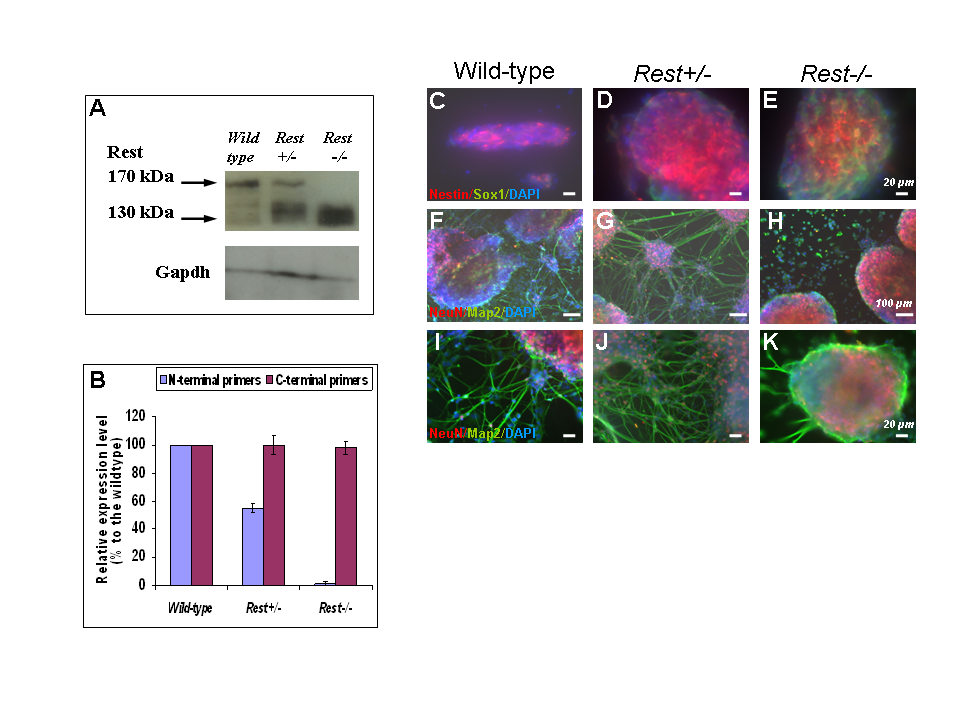

Supplement: Figure S7 — Confirmation of REST-null phenotypes in the ES cells derived from Rest knockout mice (Chen et al., 1998). (A) Rest protein expression analysed by Western blot. An alternatively spliced Rest isoform, which has a deletion in the entire exon 2 encoding the N-terminus of Rest, can be seen in ES cells derived from Rest+/− and Rest−/− mice. Gapdh was used as an internal control. (B) The expression levels of Rest in wild-type, Rest+/− and Rest−/− ES cells quantified by real-time PCR using primer sets designed against the N-ternimus and the C-terminus of Rest gene. Rest is expressed in Rest−/− ES at the wild-type level when primers against the C-terminus of Rest were used, indicating the existence of an alternatively spliced isoform which corroborated the result analysed by Western blot analysis (A). (C–E) The neural stem cells (NSCs) derived from wild-type, Rest+/− and Rest−/− ES cells were identified by Sox1 (green) and Nestin (red) from a 4-day differentiation. Nuclei were stained with DAPI (blue). The NSCs from Rest−/− ES cells show less Nestin expression in Sox1+ cells as compared to those from wild-type and Rest+/− ES cells, which is resemble to the finding in REST-null mutants (Fig. 3C and 3D). (F–H) Low power and (I–K) high power images of neurons derived from the wild-type, Rest+/− and Rest−/− ES cells. Neurons were identified by NeuN (red) and Map2 (green) after 14 days of differentiation. Nuclei were counterstained with DAPI (blue). The neurons generated from wild-type and Rest+/− ES cells show similar phenotypes to those derived from REST-100 and REST/KD-50 ES cells (Fig. 3F–G and K–L), whereas the neurons from Rest−/− to those from REST-null ES cells, which are devoid elaborated processes and migration (Fig. 3H–I and M–N). (0.66 MB TIF) [file pone.0003656.s007.tif]

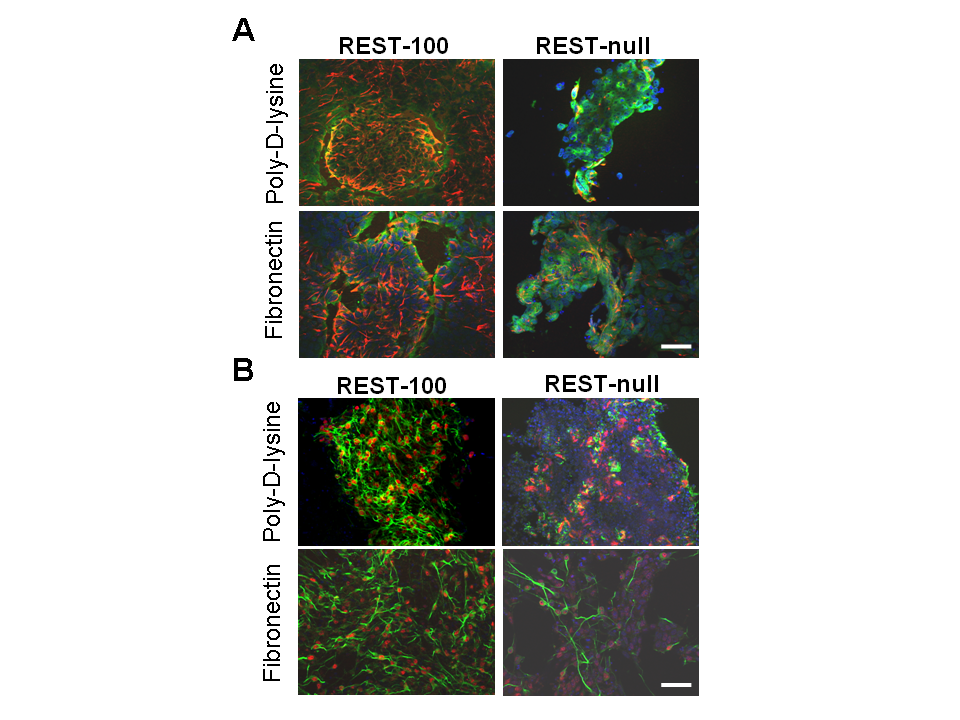

Supplement: Figure S8 — The effect of fibronectin and ploy-D-lysine on REST-null phenotypes. Control (REST-100) and REST-null ES cells were plated onto coverslips coated either with poly-D-lysine or fibronectin. The next day, cells were driven along a neural differentiation pathway in B2N27 medium. After 4 days of differentiation cells were fixed and analysed for the presence of neural stem cells by staining with Sox1 (green) and Nestin (red) (A) and the presence of neurons by staining with Map2 (green) and NeuN (red) after 14 days of differentiation (B). Nuclei were stained with DAPI (blue) (scale bar: 25 µm). All images were captured using a Zeiss fluorescence microscope equipped with an ApoTOME. (0.73 MB TIF) [file pone.0003656.s008.tif]
