# Supplementary material for: Rest-Mediated Regulation of Extracellular Matrix Is Crucial for Neural Development
Source: PLoS One. 2008 Nov 6;3(11):e3656. doi: 10.1371/journal.pone.0003656 (PMC2573962; doi:10.1371/journal.pone.0003656)
Supplement: Methods S1 — Supplemental data (0.03 MB DOC) [file pone.0003656.s009.doc]

**Supplemental data**

**Methods S1**

**Quantitative Real Time-PCR**

Primers used in this study (Table S1) were designed using the primer3 (http://frodo.wi.mit.edu/cgi-bin/primer3/primer3_www_slow.cgi) and “mfold” ([http://www.bioinfo.rpi.edu/ applications /mfold/old/dna](http://www.bioinfo.rpi.edu/applications/mfold/old/dna)) programmes to obtain amplicons using the following parameters: Tm between 58^o^C - 62^o^C, devoid of secondary structure at Tm 60^o^C and amplicon size of 50-150 bp. Real time PCR was carried out using SYBR green containing Supermix (Biorad) on a Biorad iCycler. All reactions were performed in duplicate. PCR cycle parameters were 95^o^C for 3 min, followed by 45 cycles of 95^o^C for 30 sec, 60^o^C for 30 sec, 72^o^C for 30 sec. At the end of the programme, the temperature was reduced to 50^o^C and then gradually increased by 1^o^C for 10 sec up to 96^o^C, to produce a melt curve. Gene expression was normalised to cyclophillin using the formula 1/2^∆Ct^, where ∆Ct = average unknown gene threshold cycle-average cyclophillin threshold cycle. There is no significant difference in cyclophillin expression during 18 days of differentiation (data not shown).
